# Non‐Oxidized Bare Metal Nanoparticles in Air: A Rational Approach for Large‐Scale Synthesis via Wet Chemical Process

**DOI:** 10.1002/advs.202201756

**Published:** 2022-07-22

**Authors:** Athira Thacharon, Woo‐Sung Jang, Jihyun Kim, Joohoon Kang, Young‐Min Kim, Sung Wng Kim

**Affiliations:** ^1^ Department of Energy Science Sungkyunkwan University Suwon 16419 Republic of Korea; ^2^ School of Advanced Materials Science and Engineering Sungkyunkwan University Suwon 16419 Republic of Korea

**Keywords:** electrides, negatively charged surfaces, non‐oxidized metal nanoparticles, wet chemical syntheses

## Abstract

Metal nanoparticles (MeNPs) have been used in various industrial applications, owing to their unique physical and chemical properties different from the bulk counterparts. However, the natural oxidation of MeNPs is an imminent hindrance to their widespread applications despite much research efforts to prevent it. Here, a rational approach for non‐oxidized bare MeNPs in air, which requires no additional surface passivation treatment is reported. The direct synthetic route uses the [Gd_2_C]^2+^ · 2e^−^ electride as an exceptional electron‐donating agent to reduce diverse metal precursors in alcoholic solvents. All synthesized bare Cu, Ag, and Sn nanoparticles are ultra‐stable in ambient air, exhibiting no trace of metal oxides even on their outermost atomic layer. This unique resistance to oxidation is ascribed to the accumulation of excess electrons on the surface of bare MeNPs, which originates from the spontaneous transfer of anionic electrons from the electride during the nanoparticle growth process. This approach provides not only a revolutionary scheme to obtain MeNPs with non‐passivated and non‐oxidized surfaces, but also fundamental knowledge about metal oxidation.

## Graphical abstract

A rational approach is developed to synthesize various non‐oxidized bare metal nanoparticles (MeNPs) using a wet chemical solution process. Spontaneous transfer of anionic electrons from the [Gd_2_C]^2+^ · 2e^−^ electride during reduction and growth results in excess electrons accumulated on the MeNP surface, leading to ultrahigh oxidation resistance in ambient air. This scalable approach for non‐oxidized bare MeNPs has immediate practical applications.

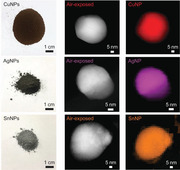

## 1 Introduction

Metal nanoparticles (MeNPs) are key ingredients for improving the physicochemical properties of diverse material systems in catalysis,^[^ ^1^ ^]^ optics,^[^ ^2^ ^]^ and electronics.^[^ ^3^ ^]^ A major challenge in developing advanced technical applications of MeNPs is the natural oxidation of bare MeNPs in air.^[^ ^4^ ^]^ In principle, most metals are inevitably oxidized when exposed to air, forming thermodynamically stable metal oxides on their surface.^[^ ^5^ ^]^ According to the Cabrera–Mott theory of metal oxidation,^[^ ^6^ ^]^ electrons released from the metal atoms react with oxygen molecules adsorbed on the surface to form oxygen anions. The metal cations then combine with the oxygen anions into surface metal oxide moieties. The natural oxidation of MeNPs in air has been resolved by the artificial manipulation of the surface structure, mainly by additional surface passivation process via post‐treatments.^[^ ^7^ ^]^ In conventional passivation processes, the MeNP surface is coated with various materials, including organic molecules,^[^ ^8^, ^9^ ^]^ polymers,^[^ ^10^ ^]^ foreign metals,^[^ ^11^ ^]^ and inorganic compounds,^[^ ^12^ ^]^ to hinder the adsorption of oxygen molecules.

The cathodic protection technique has been used for a long time to prevent corrosion by supplying excess electrons to bulky metals. The resultant negative surface charge retards the formation of metal cations.^[^ ^13^ ^]^ Although the cathodic protection technique has been widely applied to large‐scale applications in an industrial level, it was regarded as impossible to realize such negatively charged surface on the MeNPs. In a very recent study, the present authors realized the intriguing state of negatively charged surface with excess electrons by growing copper nanoparticles (CuNPs) on an exotic substrate of electride crystal.^[^ ^14^ ^]^ Electrides contain abundant anionic electrons (electron density: ≈10^22^ cm^−3^, comparable to that of free carriers in conventional metals) that are loosely bound in the structural cavities, and they have an even lower work function (≈3.0 eV)^[^ ^15^ ^]^ than that of Cu metal (4.6 eV).^[^ ^16^ ^]^ Thus, the anionic electrons are promptly transferred to the metals when they physically contact each other. The transferred excess electrons to the CuNPs from the electride are accumulated to the surface region according to the Gauss's law,^[^ ^17^ ^]^ resulting in the negatively charged Cu surface and hindering the formation of Cu cations against adsorbed oxygen molecules. This mimics the cathodic protection technique of bulky metals for the bare CuNPs and prevents the oxidation in air over several months without any additional surface manipulations.

Following this strategy, we conceived that all the MeNPs can basically have the surface‐accumulated excess electrons via the growth on electride substrates, providing an exceptional oxidation resistance in air. However, a major drawback is the small amount of non‐oxidized bare MeNPs produced and the difficulty in separating the grown MeNPs from electride substrates. Furthermore, the long‐term air stability of non‐oxidized bare MeNP powder needs to be confirmed by large‐scale analysis such as X‐ray powder diffraction (XRD) measurements. Here, we establish a wet chemical solution process as a rational approach for the large‐scale synthesis of non‐oxidized bare MeNPs having a negatively charged surface state. To reduce the diversity of metal cations in the alcoholic solvent, we select the [Gd_2_C]^2+^ · 2e^−^ electride owing to its extremely low work function (≈2.8 eV) and ferromagnetic nature, suggesting that it not only efficiently donates anionic electrons but also easily separates from non‐magnetic MeNPs using an external magnet. Another important benefit of [Gd_2_C]^2+^ · 2e^−^ electride is its chemical stability in alcoholic media. Notably, the synthesized bare CuNP, AgNP, and SnNP powders exhibit excellent air stability, showing no trace of metal oxides. Furthermore, the surface of synthesized MeNPs is very clean without any trace contamination of hydrocarbon moieties, and their metallic surface state is preserved in air.

## 2 Results and Discussions

### 2.1 Synthesis of Non‐Oxidized Bare MeNPs by Wet Chemical Solution Process

**Figure 1:**
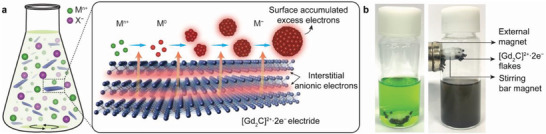
Synthesis of non‐oxidized bare MeNPs by the wet chemical solution process. a) Schematic illustration for the growth of MeNPs in alcoholic solutions containing the dissolved precursor compounds. Green and pink spheres represent metal cations and counter anions, respectively. Expanded schematic (middle panel) shows the reduction of metal cations and the growth of MeNPs. Orange arrows represent the transfer of anionic electrons from the [Gd_2_C]^2+^ · 2e^−^ electride. b) Photographs showing the color transition before and after the synthesis of CuNPs. After reaction, the [Gd_2_C]^2+^ · 2e^−^ electride flakes were removed by an external magnet.

**Figure** **1**a schematically illustrates the wet chemical solution process that uses electrides in an alcoholic solvent to produce bare MeNPs. The precursor compounds (MXs) of MeNPs were dissolved in alcoholic solvents such as anhydrous 1‐hexanol and ethanol to form metal cations (M*^n^* ^+^) and counter anions (X*^n^* ^−^), as shown in the left panel of Figure 1a. We used [Gd_2_C]^2+^ · 2e^−^ electride flakes as the reducing agent for the metal cations. A previous report^[^ ^18^ ^]^ showed that electrides generally produce solvated electrons in alcoholic solvents via alcoholysis. The solvated electrons can effectively reduce organic molecules and facilitate chemical reactions such as pinacol coupling,^[^ ^19^ ^]^ Birch reduction,^[^ ^20^ ^]^ and trifluoromethylation.^[^ ^21^ ^]^ However, [Gd_2_C]^2+^ · 2e^−^ electride is chemically stable in anhydrous 1‐hexanol and ethanol, indicating that it hardly transfers anionic electrons to the metal cations inside those solvents. This strongly suggests that charge transfer from the electride to the metal cations only occurs when the metal cations and electride flakes physically contact each other in the stirred solution. Indeed, many systems consisting of electrides and other materials in physical contact exhibit the charge transfer phenomenon driven by a difference in their work function, usually by transferring anionic electrons in the electrides.^[^ ^22^, ^23^, ^24^ ^]^

For the wet chemical solution process to produce bare MeNPs, we proved the importance of charge transfer induced by physical contact by conducting three experiments for CuNPs under different conditions. 1) Cu cations and electride flakes in the solvent were separated from each other by using a mini dialysis unit with a semipermeable membrane. As a result, hardly any CuNPs were formed, and the solution showed no color change (Figure S1, Supporting Information). 2) Without stirring the solution containing both Cu cations and electride flakes, there was only minimal contact between the Cu cations and electride flakes. This solution also showed no color change, indicating that CuNPs were not generated. 3) Stirring the solution can enhance the probability of physical contact between the Cu cations and electride flakes. This process efficiently produced the CuNPs. As shown in Figure 1b, the CuCl_2_ solution had a homogeneous green color. After the wet chemical solution process, the solution changed to black due to the formation of CuNPs.

After the reaction, the ferromagnetic [Gd_2_C]^2+^ · 2e^−^ electride flakes were easily separated using an external magnet (right panel of Figure 1b). The detailed synthesis procedures are given in the Experimental Section. The optimized conditions for various MeNPs are listed in Table S1, Supporting Information. From these results, we propose a mechanism for the generation of bare MeNPs using electrides in the wet chemical solution process. This is depicted in the expanded schematic of Figure 1a. When the metal cations contact the electride flakes, the transfer of anionic electrons occurs, resulting in the reduction of M*^n^* ^+^ to M^0^, followed by nucleation and growth to produce MeNPs. Importantly, we suggest that the transfer of anionic electrons from the electride occurs at every step, resulting in the accumulation of excess electrons at the surface of the MeNPs.

### 2.2 Surface Analysis of Non‐Oxidized Bare CuNPs

**Figure 2:**
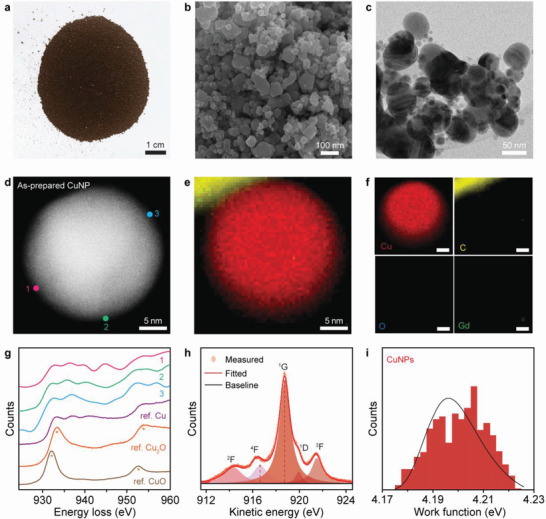
Surface analysis of the non‐oxidized bare CuNPs. a) Photograph of CuNP powder synthesized by wet chemical solution process. b) SEM image of as‐prepared CuNPs with an average size of ≈30 nm. c) TEM image of as‐prepared CuNPs. d) ADF STEM image of an as‐prepared CuNP. e) EELS elemental mapping of the ADF STEM image in (d). The mapping image shows the non‐oxidized state both in the interior and on the surface of CuNP. f) EELS elemental mapping for the Cu *L* edge (red), C *K* edge (yellow), Gd *M* edge (green), and O *K* edge (blue). Scale bars: 2 nm. g) EEL spectra obtained from points marked 1−3 in (d) at the CuNP surface, showing the metallic L_3,2_ edges. White lines of Cu oxides are denoted by asterisk (*). The white lines were not observed for the wet‐chemically synthesized CuNP. h) Cu L_3_M_45_M_45_ AES data of as‐prepared CuNPs fitted to five peaks. i) Work function histogram of the as‐prepared CuNPs measured using KPFM.

**Figure** **2**a shows an optical image of as‐prepared CuNP powder from the wet chemical solution process. The powder was obtained by drying the black solution in Figure 1b, as described in the Experimental Section. Scanning electron microscopy (SEM) and transmission electron microscopy (TEM) images of the as‐prepared CuNPs are shown in Figure 2b,c, respectively. Note that prior to all electron microscopic observations, the synthesized CuNPs were transferred without protection to the sample stage or grid, so that they were exposed to air for a few minutes. The average size of the CuNPs was ≈30 nm according to the size distribution analysis (Figure S2, Supporting Information). We analyzed the surface of as‐prepared CuNPs to verify their non‐oxidized state. Figure 2d is an annular dark‐field scanning transmission electron microscopy (ADF STEM) image of an as‐prepared CuNP. The electron energy loss spectroscopy (EELS) elemental mapping results of the as‐prepared CuNP (Figure 2e,f) clearly show no trace of oxygen at the surface. Notably, carbon‐related moieties were also not detected, indicating that the surface of the as‐prepared CuNPs was hardly contaminated even in ambient air. Furthermore, Figure 2g shows that the EEL spectra acquired from points marked 1–3 in Figure 2d match well with Cu *L*_2,3_ edges of reference Cu metal.^[^ ^25^ ^]^ We observed no white lines corresponding to cuprous oxide (Cu_2_O) or cupric oxide (CuO). In contrast, the commercial CuNPs were oxidized after exposure to air for a few minutes, as indicated by the EEL spectra of Cu oxides (Figure S3h, Supporting Information). These results highlight the exceptional oxidation resistance of the present CuNPs in a bare form without any surface passivation treatment. We further employed Auger electron spectroscopy (AES), which is a common technique for surface analysis. Considering the previous theoretical and experimental studies,^[^ ^26^, ^27^ ^]^ the measured *L*_3_ *M*_45_ *M*_45_ spectrum of the CuNPs was deconvoluted into five peaks (Figure 2h). The relative peak area ratio of ^4^F to ^1^G is 0.24, which is identical to that of metallic Cu, proving the absence of Cu oxides at the surface.

In our previous study,^[^ ^14^ ^]^ the non‐oxidized bare CuNPs synthesized by solid‐state reaction on the surface of electrides showed a lower work function than that of Cu metal. This was attributed to the surface‐accumulated excess electrons transferred from the electrides and regarded as critical evidence for the oxidation resistance in air.^[^ ^14^ ^]^ To verify whether the same phenomenon happens for the non‐oxidized bare CuNPs synthesized in this study by the wet chemical solution process, we used Kelvin probe force microscopy (KPFM) to measure the work function of CuNPs after separating them from the electride. Figure S4, Supporting Information, shows the topography and work function mapping images (the contact potential difference (CPD) values were converted to work function by calibrating the work function of the tip with highly ordered pyrolytic graphite (HOPG)). Figure 2i is a histogram of the measured work function for the wet chemically synthesized bare CuNPs. It is clear that measured values are lower than that of Cu metal (4.6 eV).^[^ ^16^ ^]^ Compared to the reported work function (≈3.2 eV) of CuNPs anchored on the surface of electrides, the average value of ≈4.2 eV in this study indicates a lower concentration of excess electrons at the surface. Nevertheless, the non‐oxidized surface of bare CuNPs synthesized here by wet chemistry is indisputable, highlighting the critical role of surface‐accumulated excess electrons in creating a negatively charged surface state of these non‐oxidized bare MeNPs. Our previous report^[^ ^14^ ^]^ revealed that the surface‐accumulated excess electrons suppress the adsorption of oxygen anions on the negatively charged metal surface, thereby prohibiting the infiltration of adsorbed oxygen into the bulk lattice and the endothermic reaction to form oxide on the negatively charged surface. Moreover, microscopic X‐ray photoelectron spectroscopy (XPS) measurement found that the CuNPs displayed a lower binding energy (932.2 eV) than that of metallic Cu (932.6 eV), also implying the existence of surface‐accumulated excess electrons (Figure S5, Supporting Information).

### 2.3 Long‐Term Air Stability of Bare CuNP Powder

**Figure 3:**
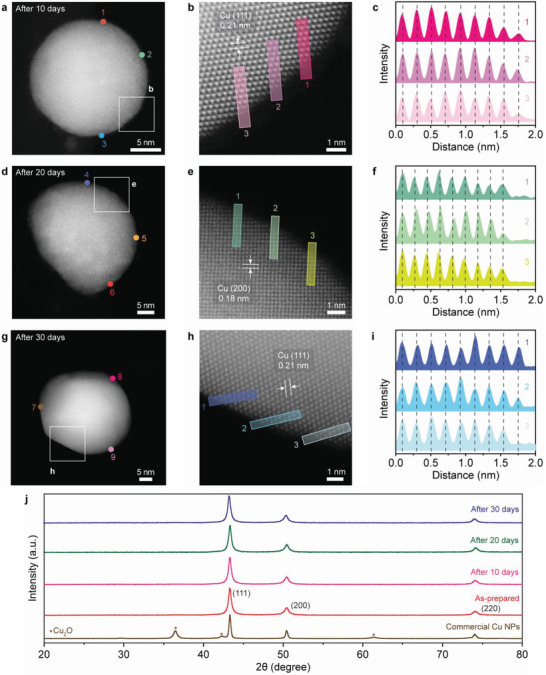
Long‐term stability of bare CuNP powder in air. a) ADF STEM image of a CuNP after 10 days of air exposure. b) Magnified HR‐STEM image of the boxed region in (a). c) Interplanar distance profile from regions marked 1−3 in (b). The interplanar distance of 0.21 nm corresponds to fcc Cu(111). d) ADF STEM image of a CuNP after 20 days of air exposure. e) Enlarged HR‐STEM image of the boxed region in (d). f) Interplanar distance profile from regions marked 1−3 in (e). The interplanar distance of 0.18 nm corresponds to fcc Cu(200). g) ADF STEM image of a CuNP after 30 days of air exposure. h) HR‐STEM image of the boxed region in (g). i) Interplanar distance profile from regions marked 1−3 in (h). The interplanar distance of 0.21 nm corresponds to fcc Cu(200). j) XRD patterns of commercial CuNPs and the bare CuNPs in the as‐prepared state and after 10, 20, and 30 days of air exposure.

The oxidation resistance of the wet chemically synthesized bare CuNP powder was investigated by exposing it to air for variable time periods. **Figure** **3**a–i shows the ADF STEM images of air‐exposed CuNPs and their atomic structures taken at 10‐day intervals. The boxed regions in Figure 3a,d,g are respectively magnified in Figure 3b,e,h to show the surface atomic arrangement. From the high‐resolution ADF STEM observations, which avoid the Fresnel fringes at the surfaces, it is clear that the periodic arrangements with an interplanar distance of 0.21 nm (Figure 3c,i) and 0.18 nm (Figure 3f) in the (111) and (200) planes are preserved up to the outermost atomic layer, indicating the absence of Cu oxides in the surface area. In contrast, the commercial CuNPs were heavily oxidized after air exposure for a few minutes, as easily distinguished in Figure S3f, Supporting Information, where the interplanar distance of 0.25 nm corresponds to the (111) plane of Cu_2_O.^[^ ^28^ ^]^ Moreover, the EELS data acquired from points 1–9 in the surface areas of Figure 3a,d,g show no white lines of Cu_2_O or CuO (Figure S6, Supporting Information), proving that the wet‐chemically synthesized bare CuNPs were non‐oxidized. STEM‐EELS mapping results of the air‐exposed bare CuNPs are also provided in Figure S7, Supporting Information, where no oxygen or carbon elements were detected at the surface, strongly indicating that the oxidation‐free metallic surface was maintained for at least 30 days in air. In addition, the XPS spectra of air‐exposed bare CuNPs showed a clear negative shift in the binding energy of Cu 2p, strongly suggesting that the surface‐accumulated excess electrons remained after air exposure providing an excellent oxidation resistance in air (Figure S5, Supporting Information).

Furthermore, we confirmed the long‐term air stability for a large amount of non‐oxidized CuNP powder. Figure 3j shows the XRD patterns of the as‐prepared and air‐exposed bare CuNP powder at 10‐day intervals, as well as the commercial CuNP powder for comparison. The commercial CuNP powder showed distinct diffraction peaks of Cu_2_O. In contrast, no diffraction peaks of Cu oxides were detected in any of the bare CuNP powder. Considering all these results of air‐exposed CuNPs and their powder, it is evident that our large‐scale synthesis via the wet chemical solution process successfully produced non‐oxidized bare CuNP powder. This is beneficial for the practical applications of bare CuNPs without additional surface passivating processes. Additionally, we checked the reusability of the electride flakes. As discussed earlier, these flakes are ferromagnetic and thus can be easily and effectively separated by using an external magnet after the reaction is complete (Figure 1b). Optical images and XRD patterns of the electride flakes before and after the reaction are shown in Figures S8 and S9, Supporting Information, respectively. No degradation of the crystal structure was observed in the remaining electride flakes after the reaction. Notably, when these flakes were reapplied in a continuous reaction, non‐oxidized bare CuNPs were also successfully produced, as confirmed by the EEL spectra at the surface (Figure S10, Supporting Information). However, some anionic electrons in the electrides were consumed during the reduction of anhydrous CuCl_2_ precursor and the generation of surface‐accumulated excess electrons, suggesting that the electride was partially decomposed. Indeed, after the reaction and washing procedures, the electride lost ≈30% of its original weight, which is consistent with the numerical calculation (described in the Experimental Section) that one third of the anionic electrons can be consumed under the present reaction conditions. For industrial applications of our wet chemical solution process, it is necessary to develop an optimized condition with a larger amount of electride.

### 2.4 Rational Strategy for Other Non‐Oxidized Bare MeNPs in Air

**Figure 4:**
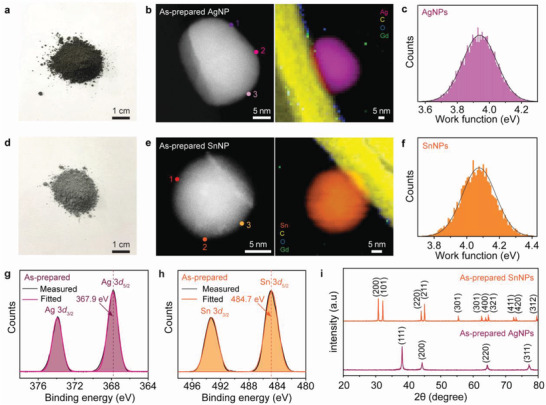
Rational strategy for preparing non‐oxidized bare MeNPs in air. a) Optical image of AgNP powder synthesized by wet chemical solution process. b) ADF STEM and EELS mapping image of as‐prepared AgNPs: the Ag *M* edge (pink), C *K* edge (yellow), Gd *M* edge (green), and O *K* edge (blue). c) Histogram of work function for as‐prepared AgNPs measured by KPFM (average value: ≈3.9 eV). d) Optical image of SnNP powder synthesized by wet chemical solution process. e) ADF STEM and EELS mapping image of as‐prepared SnNPs: the Sn *M* edge (orange), C *K* edge (yellow), Gd *M* edge (green), and O *K* edge (blue). f) Histogram of work function for as‐prepared SnNPs measured by KPFM (average value: ≈4.1 eV). g) Ag 3d XPS spectra of as‐prepared AgNPs. A negative shift in the binding energy of the Ag 3d_5/2_ peak (367.9 eV) directly implies a high electron density on the surface. h) Sn 3d XPS spectra of as‐prepared SnNPs. A negative shift in the binding energy (484.7 eV) was observed for Sn 3d_5/2_ compared to Sn metal. i) XRD patterns of as‐prepared AgNP and SnNP powders without peaks of metal oxides.

Next, the established wet chemical solution process for non‐oxidized bare CuNPs was extended to other bare MeNPs (Me = Ag and Sn). The detailed reaction conditions are provided in Table S1, Supporting Information and Experimental Section. It should be noted that both precursor compounds, AgNO_3_ for Ag and Sn(OAc)_2_ for Sn, contain oxygen, which can cause oxidation of AgNPs and SnNPs in the solvents. **Figure** **4** summarizes the results of synthesized bare AgNPs and SnNPs. Optical images of the wet‐chemically synthesized AgNP and SnNP powders (Figure 4a,d) exhibit the corresponding typical color. As confirmed for the non‐oxidized bare CuNP powder, ADF STEM and EELS mapping images of the as‐prepared AgNPs and SnNPs also demonstrated that both particle surfaces contained no trace of oxides (Figure 4b,e). The measured EEL spectra of both NPs at points marked 1–3 indicate that the metallic states of Ag *M* and Sn *M* edges were maintained at each surface, which is consistent with those of the reference Ag^[^ ^29^ ^]^ and Sn^[^ ^30^ ^]^ metals (Figure S11, Supporting Information). Histograms obtained from KPFM measurements show lower work functions for the as‐prepared AgNPs (≈3.9 eV on average) and SnNPs (≈4.1 eV on average) compared to those of Ag (≈4.3 eV)^[^ ^31^ ^]^ and Sn (≈4.4 eV) metals,^[^ ^32^ ^]^ providing a direct evidence of the surface state with excess electrons (Figure 4c,f). Furthermore, XPS measurements of the as‐prepared AgNP and SnNP powders were conducted to verify the presence of surface‐accumulated excess electrons, which should result in a negatively charged state with a lower binding energy. As shown in Figure 4g,h, the measured binding energies of Ag 3d_5/2_ (367.9 eV) and Sn 3d_5/2_ (484.7 eV) are obviously lower than those of metallic Ag (368.2 eV)^[^ ^33^ ^]^ and Sn (485.0 eV),^[^ ^34^ ^]^ respectively. In addition, the XRD patterns of as‐prepared AgNP and SnNP powders also showed only the diffraction peaks of Ag and Sn metals without any trace of oxide formation (Figure 4i).

**Figure 5:**
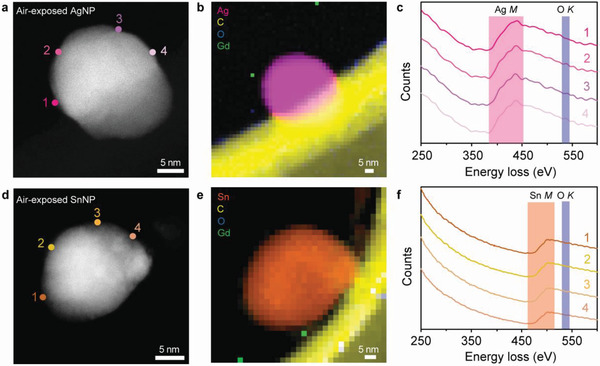
Air stability of non‐oxidized bare AgNPs and SnNPs. a) ADF STEM image of an air‐exposed AgNP for 30 days. b) EELS mapping image of (a): the Ag *M* edge (pink), C *K* edge (yellow), Gd *M* edge (green), and O *K* edge (blue). c) EEL spectra obtained from surface points marked 1−4 in (a). d) ADF STEM image of an air‐exposed SnNP for 1 day. e) EELS mapping image of (d): the Sn *M* edge (orange), C *K* edge (yellow), Gd *M* edge (green), and O *K* edge (blue). f) EEL spectra obtained from surface points marked 1−4 in (d). The EEL mapping image and EEL spectra show metallic *M* edges of air‐exposed Ag and SnNPs without any detectable O *K* signal.

These results clearly indicate the non‐oxidized behavior of bare MeNPs synthesized by the wet chemical solution process, despite the use of oxygen containing solutions. This emphasizes the role of surface‐accumulated excess electrons in preventing the adsorption of oxygen species on the metallic surfaces of the bare MeNPs. Finally, we tested the oxidation resistance of bare MeNPs in air. **Figure** **5** shows the ADF STEM and EELS mapping images and the EEL spectra of air‐exposed AgNPs and SnNPs taken after 30 days and 1 day, respectively. Similar to their as‐prepared counterparts, no metal oxides were formed at the surfaces of air‐exposed NPs, as evidenced by the absence of the O *K* edge at ≈532 eV in the EEL spectra obtained at surface points 1−4 in Figure 5a,d. Furthermore, XPS measurements of air‐exposed AgNPs and SnNPs confirmed that there was no change in the binding energy compared to the as‐prepared samples, indicating the retention of surface‐accumulated excess electrons that prevented the bare MeNPs from air oxidation (Figure S12, Supporting Information).

## 3 Conclusion

We established a rational approach for the large‐scale synthesis of bare non‐oxidized MeNPs using a wet chemical solution process. Importantly, the spontaneous charge transfer of anionic electrons from the [Gd_2_C]^2+^ · 2e^−^ electride occurs during the nucleation and growth of MeNPs in the solution, leading to a negatively charged metal surface with accumulated excess electrons. Atomic‐scale microscopy and spectroscopy measurements highlighted that the synthesized bare CuNPs, AgNPs, and SnNPs were remarkably stable in ambient air, as evidenced by the absence of metal oxides at the surface. The XRD patterns of the synthesized MeNPs and the reusability of the electride flakes demonstrated the practical applicability of the proposed wet chemical solution process for mass producing non‐oxidized bare MeNPs. Our protocol will facilitate the preparation of other bare MeNPs with ultra‐high resistance against oxidation in air, as well as allow the shortened process for their large‐scale synthesis by excluding the additional surface passivation treatments.

## 4 Experimental Section

### Synthesis of [Gd_2_C]^2+^ · 2e^−^ Electride

[Gd_2_C]^2+^ · 2e^−^ electride was synthesized according to a previously reported protocol.^[^ ^35^ ^]^ Arc melting was applied to Gd metal pieces (Alfa Aesar, 99.9%) and graphite (LTS Chemicals) in a molar ratio of 2:1. A process of cooling and re‐melting was repeated to obtain high‐quality single‐phase [Gd_2_C]^2+^ · 2e^−^ electride and ensure homogeneity. After the melting process, the polycrystalline [Gd_2_C]^2+^ · 2e^−^ ingot was quickly moved into a glove box filled with high‐purity Ar gas (99.999%). The fresh ingot was crushed into millimeter‐sized flakes for the wet chemical synthesis of MeNPs.

### Synthesis of CuNPs

First, anhydrous CuCl_2_ (5 mmol) was dissolved in hexanol (50 mL) by magnetic stirring (30 min). Next, the [Gd_2_C]^2+^ · 2e^−^ electride flakes (10 mmol) were added to the CuCl_2_ solution and stirred using a magnetic bar at 80 °C for 12 h. The molar ratio of CuCl_2_:[Gd_2_C]^2+^ · 2e^−^ was 1:2. After the reaction, the electride flakes were separated from the CuNP dispersion using an external magnet, without any post‐treatment. Finally, the solution was tip sonicated (10 min) and centrifuged (9000 rpm for 20 min) several times to obtain a blackish‐brown residue, which was then dried in a vacuum chamber. When 5 mm of CuCl_2_ (0.67 g) is reduced by 10 mm, of [Gd_2_C]^2+^ · 2e^−^ electride (3.26 g) with a yield of CuNPs is ≈80% (Table S1, Supporting Information), the number of electrons of 4.82 × 10^21^ is required, which is nearly one third of total number of electrons (12.04 × 10^21^) in the used [Gd_2_C]^2+^ · 2e^−^ electride. As a reference, commercial CuNPs with a diameter of ≈40 nm and capped with polyvinylpyrrolidone were purchased from US Research Nanomaterials Inc.^[^ ^36^ ^]^

### Synthesis of AgNPs

First, anhydrous AgNO_3_ (5 mmol) was dissolved in anhydrous ethanol (50 mL) by magnetic stirring (30 min) in a dark amber vial. Next, [Gd_2_C]^2+^ · 2e^−^ electride flakes (10 mmol) were added to the AgNO_3_ solution and stirred using a magnetic bar at 70 °C for 12 h. After the reaction, the electride flakes were separated from the AgNP dispersion using an external magnet. Finally, the solution was tip‐sonicated (10 min) and centrifuged (9000 rpm for 20 min) several times to obtain a black residue, which was dried in a vacuum chamber.

### Synthesis of SnNPs

First, C_4_H_6_O_4_Sn (5 mmol) was dissolved in anhydrous 1‐hexanol (50 mL) by magnetic stirring (30 min) in a dark amber vial. Then, [Gd_2_C]^2+^ · 2e^−^ electride flakes (10 mmol) were added to the Sn(OAc)_2_ solution and stirred using a magnetic bar at 25 °C for 12 h. After the reaction, the electride flakes were separated from the SnNP dispersion using an external magnet. Finally, the solution was tip‐sonicated (10 min) and centrifuged (9000 rpm for 20 min) several times to obtain a gray‐colored residue, which was dried in a vacuum chamber.

### Characterization

The morphology of MeNPs was analyzed using field‐emission SEM (JEOL, JSM‐7600F). HR‐TEM, STEM, and EELS measurements were carried out using probe Cs‐corrected JEM‐ARM200CF (JEOL Ltd.) operated at 200 kV. All samples were dispersed in *n*‐heptane, applied to a 300‐mesh gold holey carbon grid (TED PELLA INC.) in an argon‐filled glovebox, and dried in a vacuum chamber. For EELS mapping, the sample drift during acquisition was corrected by tracking the position of the reference particle assigned at the beginning of the acquisition. EELS measurements were performed in the STEM mode using the same microscope equipped with a Gatan imaging filter (GIF) detector (Gatan Quantum 965 ER). Spectroscopic measurements were carried out at an energy dispersion of 0.5 eV per channel, and the energy resolution at the zero‐loss peak was 0.9 eV. XPS analysis of MeNPs and AES analysis of Cu L_3_M_45_M_45_ were performed using an R4000 spectrometer (VG Scienta) with an Al K*α* X‐ray source (1486.7 eV). The XPS and AES spectra were calibrated using the Au 4f peak at 84.0 eV as the reference. An atomic force microscope (MFP‐3D AFM, Asylum Research) equipped with a sealed electrochemistry cell filled with argon gas was used to measure the surface work function of the samples deposited on an Au‐coated Si substrate. KPFM measurements were carried out using a Ti/Ir‐coated Si probe (ASYELEC.01‐R2, Asylum Research) with a resonance frequency of 75 kHz and a force constant of 2.8 N m^−1^. A highly oriented pyrolytic graphene (HOPG) reference sample with a well‐known work function was used to calibrate the work function of the tip.^[^ ^37^ ^]^ During the KPFM scanning process, the scan rate and set point were 0.8 Hz and 0.5 V, respectively. The tip was lifted 30 nm from the sample, and an a.c. voltage (*V*_a.c._ = 1 V) was applied to the tip. XRD measurements of the CuNPs and [Gd_2_C]^2+^ · 2e^−^ electride were carried out using an X‐ray diffractometer (Rigaku Smartlab) with monochromatic Cu K*α*1 line (8.04 keV). A plastic dome‐type stage filled with argon gas was used to investigate the crystal structure of [Gd_2_C]^2+^ · 2e^−^ electride to avoid oxidation during analysis.

### Statistical Analysis

All statistical analyses of the STEM‐EELS spectra, interplanar distance profiles, XPS and AES spectra, KPFM data, XRD data, and SEM particle size distribution data were performed using the Origin software.

## Conflict of Interest

The authors declare no conflict of interest.

## Supporting information

Supporting Information

## Data Availability

The data that support the findings of this study are available from the corresponding author upon reasonable request.
