# Supplementary material for: Non‐Oxidized Bare Metal Nanoparticles in Air: A Rational Approach for Large‐Scale Synthesis via Wet Chemical Process
Source: Adv Sci (Weinh). 2022 Jul 22;9(26):2201756. doi: 10.1002/advs.202201756 (PMC9475554; doi:10.1002/advs.202201756)
Supplement: Supplementary file 1 — Supporting Information [file ADVS-9-2201756-s001.pdf]

ADVANCED SCIENCE
Open Access

Supporting Information

for *Adv. Sci.*, DOI 10.1002/advs.202201756

Non-Oxidized Bare Metal Nanoparticles in Air: A Rational Approach for Large-Scale Synthesis via Wet Chemical Process

Athira Thacharon, Woo-Sung Jang, Jihyun Kim, Joohoon Kang, Young-Min Kim* and Sung Wng Kim**

Supporting Information

Non-Oxidized Bare Metal Nanoparticles in Air: A Rational Approach for Large-Scale Synthesis via Wet Chemical Process

Athira Thacharon, Woo-Sung Jang, Jihyun Kim, Joohoon Kang, Young-Min Kim*, Sung Wng Kim**

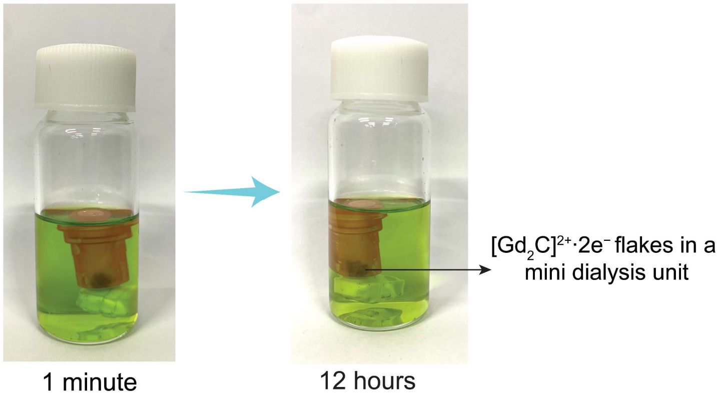

Figure S1. CuNPs synthesis by non-contact method using a mini dialysis unit with a semipermeable membrane.

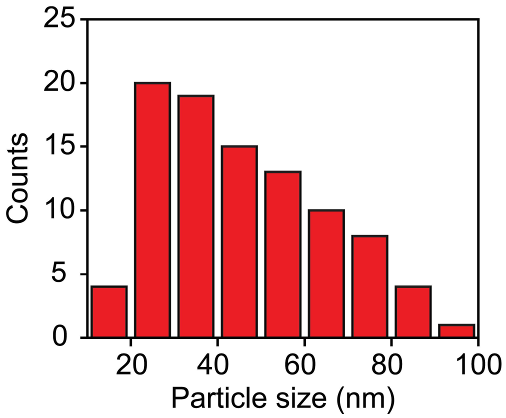

Figure S2. Size distribution profile of CuNPs from SEM.

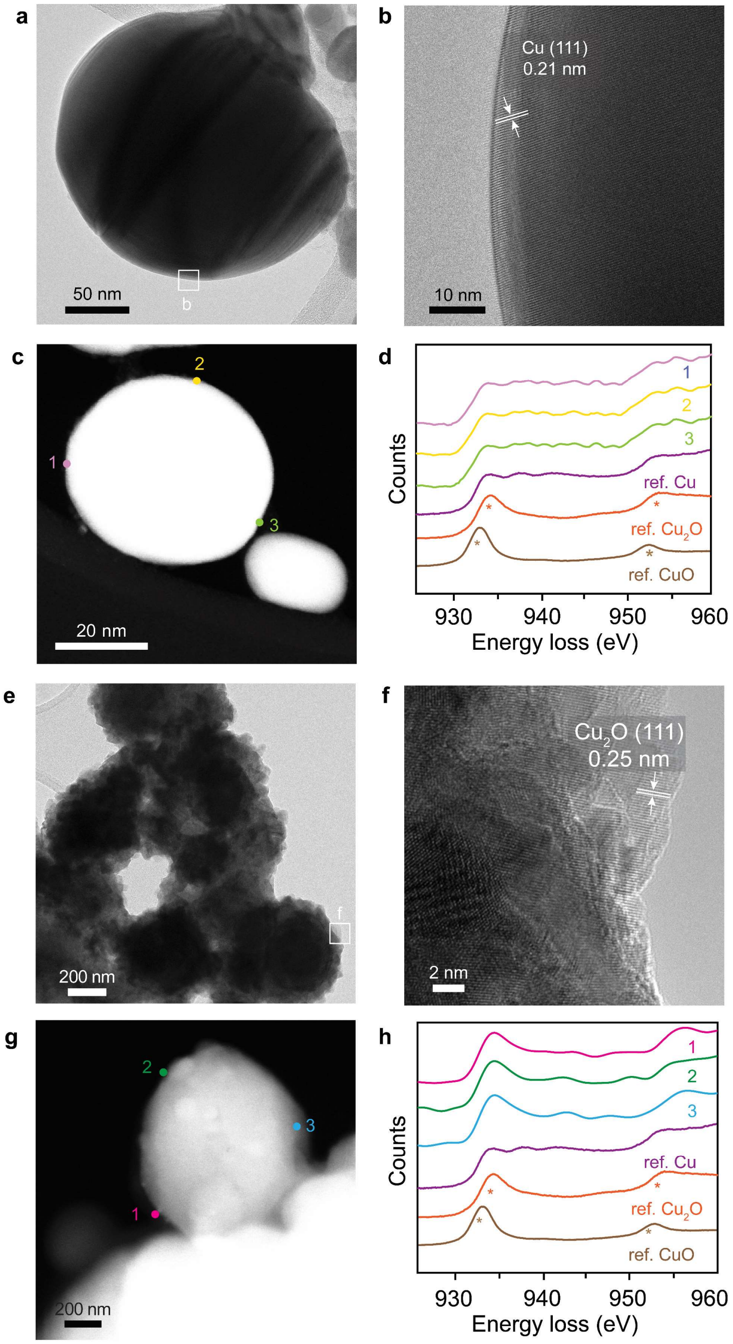

Figure S3. Comparison of wet-chemically synthesized and commercial CuNPs. a) TEM image of wet-chemically synthesized CuNPs. b) HR–TEM image of wet-chemically synthesized CuNP. Interplanar distance of 0.21 nm corresponding to Cu(111) plane. c) STEM image of wet-chemically synthesized CuNP. d) EEL spectra obtained from the marked points 1–3 from c clearly shows metallic $L_{3,2}$ edge indicating no oxidation. e) TEM image of commercial CuNPs. f) HR–TEM image of commercial CuNPs. Interplanar distance of 0.25

nm corresponding to Cu_2O (111) plane. g) STEM image of commercial CuNP. h) EEL spectra obtained from the marked points 1−3 in g clearly shows white lines of Cu_2O, indicating the occurrence of oxidation.

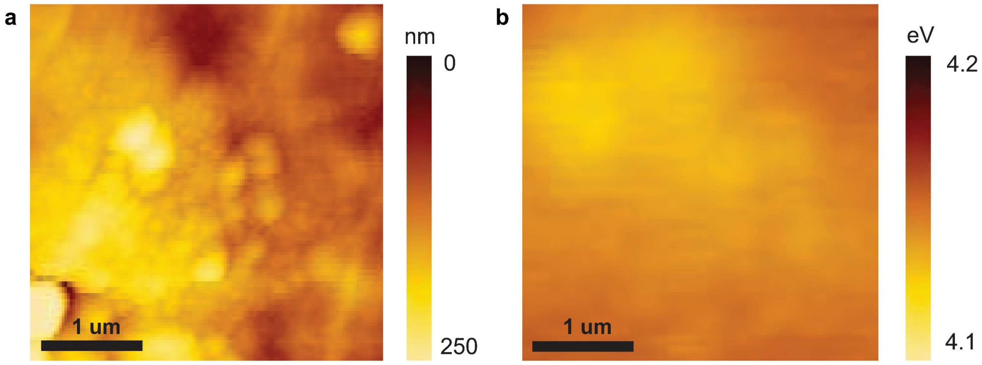

Figure S4. a) Topography and b) work function mapping image of as-prepared CuNPs.

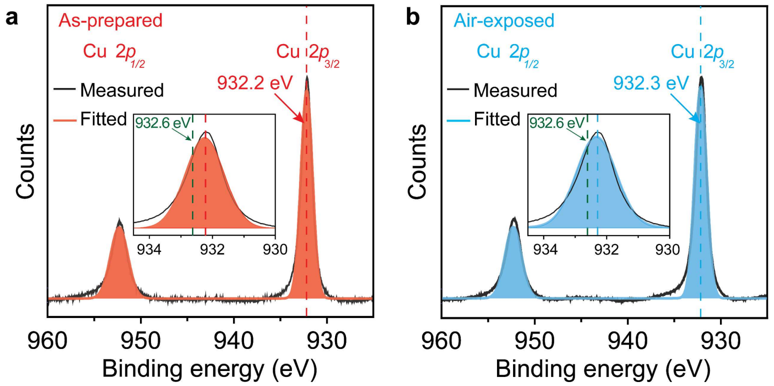

Figure S5. XPS of as-prepared and air-exposed CuNPs (15 days). Insets show the negative shift in binding energy of wet-chemically synthesized CuNPs from metallic Cu (green dotted line, 932.6 eV).

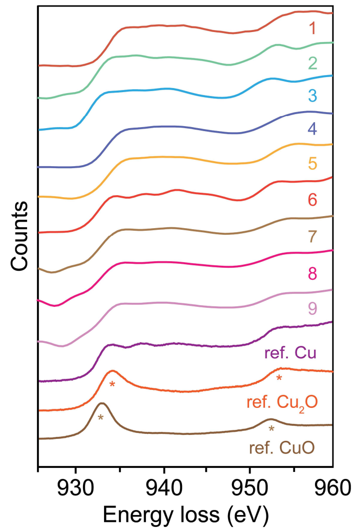

Figure S6. EELS data obtained from surface points 1–9 in the STEM images shown in Figure 3a, d, and g of air-exposed CuNPs.

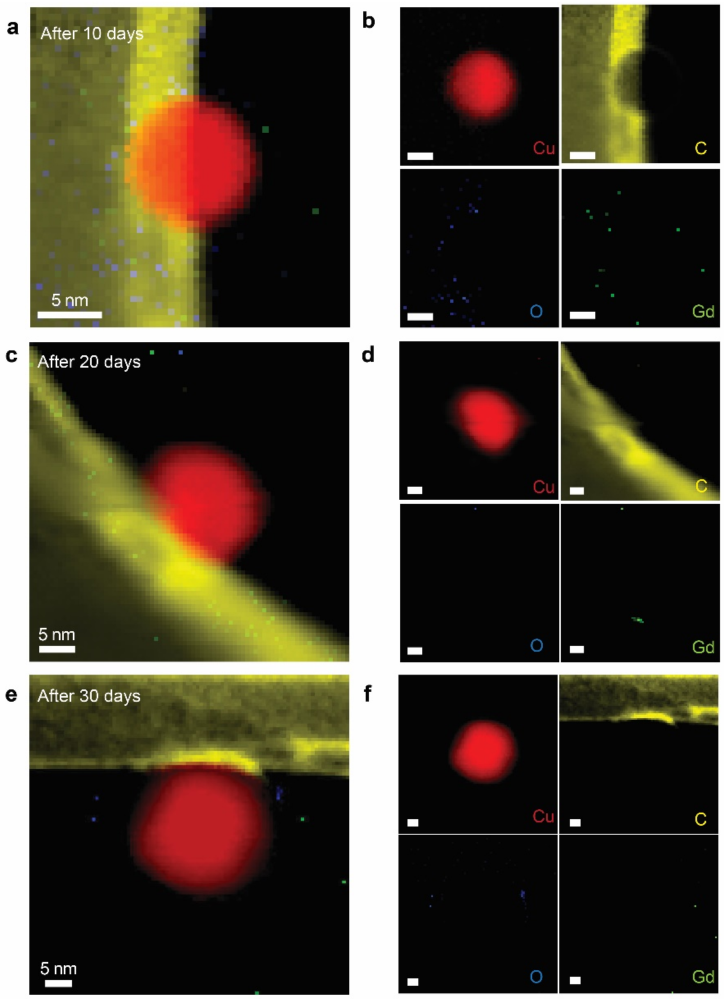

Figure S7. a–f) EELS mapping distribution of air-exposed CuNPs for 10, 20, and 30 days respectively. Cu *L* edge (red), C *K* edge (yellow) Gd *M* edge (green), O *K* edge (blue). Scale bars in corresponds to 2nm.

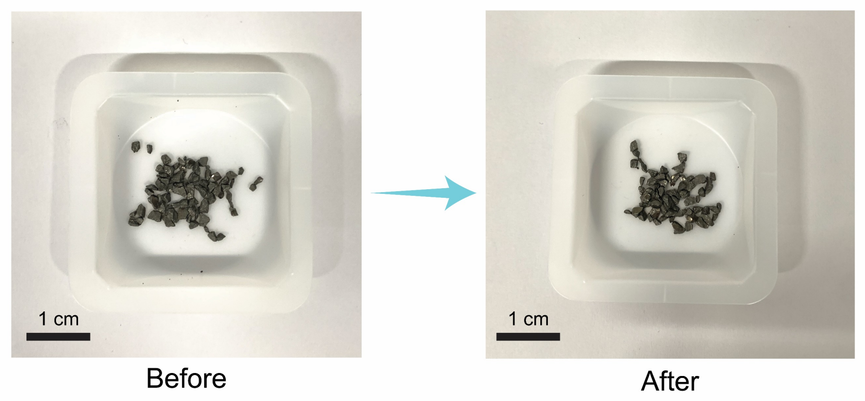

Figure S8. Optical image of $[Gd_2C]^{2+}\cdot 2e^{-}$ electride flakes used for wet chemical synthesis of CuNPs; before (left) and after (right) reaction.

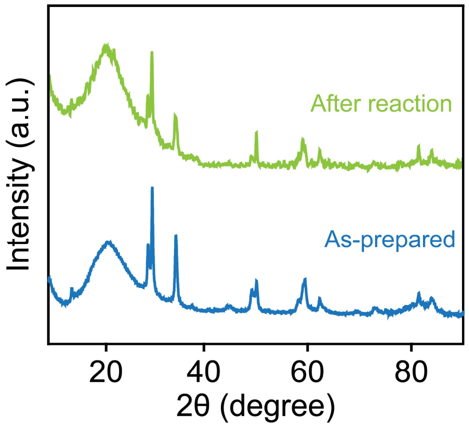

Figure S9. Powder XRD of $[Gd_2C]^{2+}\cdot 2e^{-}$ electride flakes; as-prepared for a reaction (bottom) and after reaction (top) producing CuNPs via wet chemical solution process.

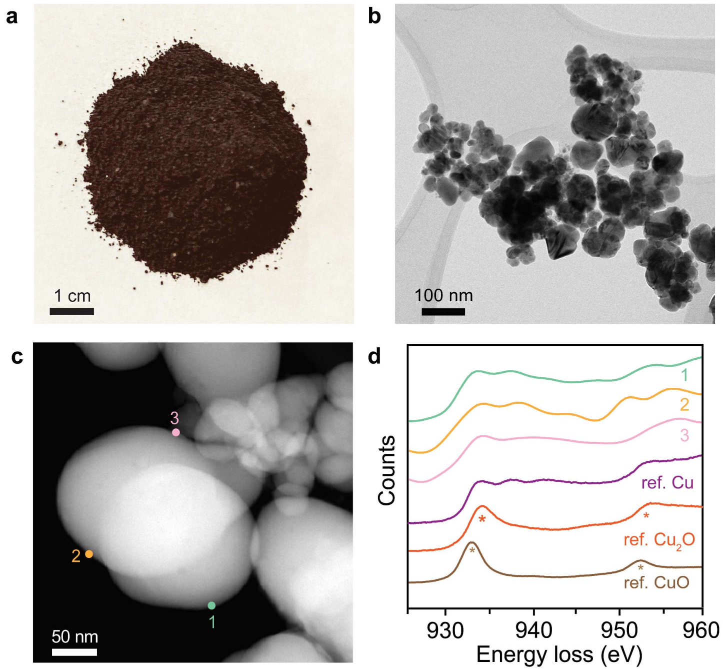

Figure S10. a) Photograph of CuNP powder synthesized by the reused $[Gd_2C]^{2+}\cdot 2e^-$ electride flakes. b) TEM image of CuNPs synthesized using the reused $[Gd_2C]^{2+}\cdot 2e^-$ electride. c) STEM image of CuNPs synthesized using the reused $[Gd_2C]^{2+}\cdot 2e^-$ electride. d) EELS data of CuNPs synthesized using the reused $[Gd_2C]^{2+}\cdot 2e^-$ showing metallic $L_{3,2}$ edge from marked points 1–3 in c.

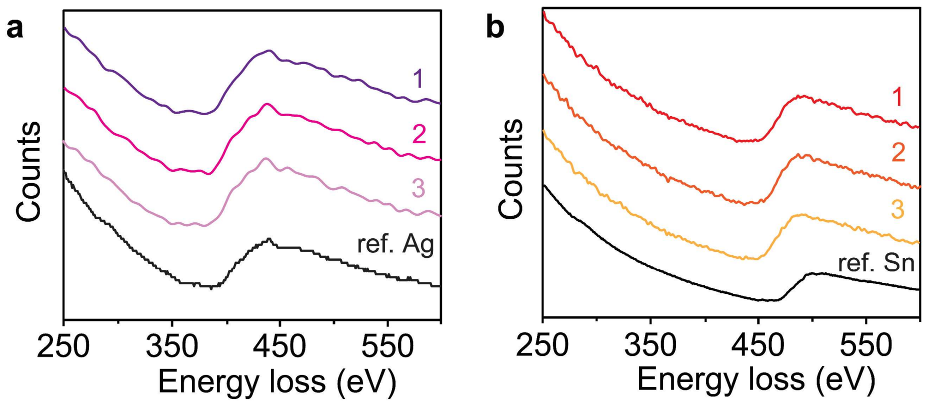

Figure S11. EELS data obtained from surface points 1–3 in the STEM images shown in Figures 4b and 4e of as-prepared AgNPs and SnNPs, respectively.

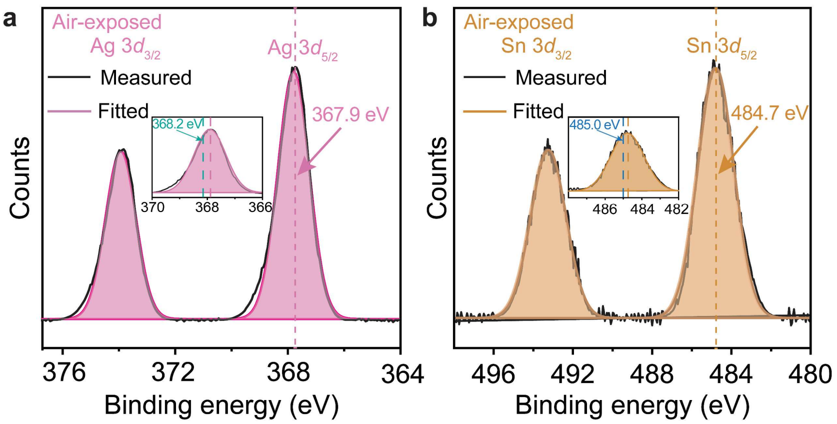

Figure S12. XPS of air-exposed Ag (15 days) and Sn (1 day) NPs. Insets show the negative shift in binding energy of air-exposed Ag (367.9 eV) and Sn (484.7 eV) NPs from metallic Ag (cyan dotted line, 368.2 eV) and Sn (blue dotted line, 485.0 eV).

Table S1. Optimization of syntheses of various MeNPs by wet chemical solution process.

**Precursor**	**Reducing agent**	**Solvent**	**Temperature [°C]**	**Stirring [rpm]**	**Time [h]**	**Yield**
$CuCl_2$	$[Gd_2C]^{2+}\cdot 2e^-$	Hexanol	25	0	12	N.R.
$CuCl_2$	$[Gd_2C]^{2+}\cdot 2e^-$	Hexanol	25	450	12	N.R.
$CuCl_2$	$[Gd_2C]^{2+}\cdot 2e^-$	Hexanol	80	0	12	N.R.
$CuCl_2$	$[Gd_2C]^{2+}\cdot 2e^-$	Hexanol	80	450	12	~80 %
$AgNO_3$	$[Gd_2C]^{2+}\cdot 2e^-$	Ethanol	70	0	12	N.R.
$AgNO_3$	$[Gd_2C]^{2+}\cdot 2e^-$	Ethanol	70	450	12	~80 %
$Sn(OAc)_2$	$[Gd_2C]^{2+}\cdot 2e^-$	Hexanol	25	0	12	N.R.
$Sn(OAc)_2$	$[Gd_2C]^{2+}\cdot 2e^-$	Hexanol	25	450	12	~78 %

(N.R. : no reaction occurred)
